# How to evaluate the acquisition of clinical skills at medical school: a tough question

**DOI:** 10.1590/S1516-31802004000100001

**Published:** 2004-01-08

Assessment of medical students' knowledge is a very difficult issue. Many types of student evaluation are possible, including written longitudinal tests (the so-called "progress tests" or "evolution tests"), module-related written tests and the Objective Structured Clinical Examination (OSCE).

The OSCE was introduced into some medical schools in the 1970s and a lot of information is now available regarding its reliability, validity and effectiveness as an assessment tool.^1-3^ It was created to enable better assessment and quantification of clinical skills acquisition by medical students. In the majority of medical schools, clinical skills are taught to small groups of 6-15 students. This small number of students allows better assessment of the student by his/her tutor. However, even in small groups, it is very difficult for the tutor to evaluate each student individually.^4^ The OSCE exam was created to individualize this evaluation. The student's behavior, attitude and decisions regarding a clinical situation are analyzed at various stations, each requiring specific tasks, including history-taking, physical examination and therapeutic skills, with an observer inside the room at most of the stations.

The only major experience so far in Brazil with this form of student assessment has taken place at the University of São Paulo School of Medicine, at the Ribeirão Preto city campus. The OSCE was applied to medical students in Ribeirão Preto for three consecutive calendar years, in their 5^th^ semester, i.e. after the introductory course on basic clinical skills. The results from this experience are analyzed in this issue of the *Journal* by the same group that was responsible for OSCE application.^5^

The results are very intriguing. In the first application of the OSCE, 48% of the students criticized many managerial aspects. Improvement in the way it was applied reduced the student complaint rate to just 5%. However, the students still reported difficulties with time management and stress control. These were, respectively, 70% and 70% in the first application of the OSCE, and 40% and 75% in the third year of application, i.e. after the introduction of a lot of modifications, including more time for solving the clinical situations.

With regard to the students' difficulties, one possible explanation is that, throughout students' academic lives before university, and even at university, they are trained in cognitive forms of evaluation. It becomes very embarrassing when his/ her expertise in analyzing a clinical situation is assessed by an observer inside the room verifying if he/she is doing the right thing. The presence of an observer may, in these cases, be a stressful situation. It is possible that, if a form of evaluation using the same structure as in the OSCE were to be introduced in the first semester of a medical school course, with several stations and an observer posted in some or all of the stations, the stress factor would be reduced.

The other possible explanation is that we are not teaching students exactly how much time they have for performing their tasks. Maybe it is time to point out that students do not have all the time in the world to do simple things. It is obvious that the length of time proposed has to be adequate for a younger student to do his or her tasks. Nonetheless, students should at least have a limit on the time available for performing their tasks that is set as a goal to be reached after a clinical skills course.

With regard to the teachers in the university staff, one possible point is that, for an OSCE to be implemented, a lot of people need to work hard for many months. Most of the lecturers in a medical school divide their time between teaching, research and clinical practice. Thus, if we want to apply better forms of evaluation, such as the OSCE, we need more people teaching and better clerical support.

It is therefore time for medical schools in Brazil to create a better structure for evaluating and analyzing the performance of their students, with more medical schools involved in this task and more time made available for doing this. If not, the results will be the same as found in Ribeirão Preto. There, despite the remarkable quantity of time and effort expended, the impact of the OSCE was very limited, with low participation by other faculty members who were not involved in the project.
